# Sex-specific incidence and temporal trends in solid tumours in young people from Northern England, 1968–2005

**DOI:** 10.1186/1471-2407-8-89

**Published:** 2008-04-03

**Authors:** Brooke L Magnanti, M Tevfik Dorak, Louise Parker, Alan W Craft, Peter W James, Richard JQ McNally

**Affiliations:** 1 School of Clinical Medical Sciences (Child Health), Newcastle University, Sir James Spence Institute, Royal Victoria Infirmary, Newcastle upon Tyne NE1 4LP, UK; 2 IWK Health Centre, Dalhousie University, Halifax, Nova Scotia B3K 6R8, Canada; 3 School of Clinical Medical Sciences (Child Health) and Institute of Health and Society, Newcastle University, Sir James Spence Institute, Royal Victoria Infirmary, Newcastle upon Tyne NE1 4LP, UK

## Abstract

**Background:**

This study examined sex-specific patterns and temporal trends in the incidence of solid tumours in the Northern Region of England from 1968 to 2005. This updates earlier analyses from the region where sex was not considered in depth. Sex-specific analyses were carried out to determine whether sex differences might provide clues to aetiology.

**Methods:**

Details of 3576 cases, aged 0–24 years, were obtained from a specialist population-based cancer registry. There were 1843 males (886 aged 0–14 years and 957 aged 15–24 years) and 1733 females (791 aged 0–14 years and 942 aged 15–24 years). Age-standardized incidence rates (per million population) were calculated. Linear regression was used to analyze temporal trends in incidence and annual percentage changes were estimated. Analyses were stratified by sex and by age-group.

**Results:**

There were marked differences in incidence patterns and trends between males and females and also between age-groups. For males central nervous system (CNS) tumours formed the largest proportion of under-15 cases and germ cell tumours was the largest group in the 15–24's, whilst for females CNS tumours dominated in the under-15's and carcinomas in the older group. For 0–14 year olds there were male-specific increases in the incidence of rhabdomyosarcoma (2.4% per annum; 95% CI: 0.2%–4.5%) and non-melanotic skin cancer (9.6%; 95% CI: 0.0%–19.2%) and female-specific increases for sympathetic nervous system tumours (2.2%; 95% CI: 0.4%–3.9%), gonadal germ cell tumours (8.6%; 95% CI: 4.3%–12.9%) and non-gonadal germ cell tumours (5.4%; 95% CI: 2.8%–7.9%). For 15–24 year olds, there were male-specific increases in gonadal germ cell tumours (1.9%; 95% CI: 0.3%–3.4%), non-gonadal germ cell tumours (4.4%; 95% CI: 1.1%–7.7%) and non-melanotic skin cancer (4.7%; 95% CI: 0.5%–8.9%) and female-specific increases for osteosarcoma (3.5%; 95% CI: 0.5%–6.5%), thyroid cancer (2.8%; 95% CI: 0.1%–5.6%) and melanoma (4.6%; 95% CI: 2.2%–7.1%).

**Conclusion:**

This study has highlighted notable differences between the sexes in incidence patterns and trends for solid tumours. Some of these sex-specific differences could have been obscured if males and females had been analysed together. Furthermore, they suggest aetiological differences or differential susceptibility to environmental factors between males and females.

## Background

Sex-related differences in incidence of childhood cancer are well-established and consistent worldwide [1-4]. Sex of the patient can also play a role in efficiency of diagnosis and treatment [5]. Therefore it is important to include both male and female results in reported trends, even for childhood and young persons' cancers.

Unfortunately this has not always been done. For instance, a recent paper reported solid tumour rates in north-west Italy over a similar time period of 1967–2001 [6]. Sex ratios have been given where the incidence rates were reported. However, in calculating the annual average percentage rate change the sexes have been combined. Other papers have also reported overall and not sex-specific results [7]. This may have obscured sex-specific temporal trends, especially over an extended time period.

Previous studies from the Northern Region of England have assessed the incidence of solid tumours diagnosed in 0 – 24 year olds during the periods 1968 – 1982 and 1968 – 1995 [8,9]. Another study from the Northern region has focused specifically on the 15–24 age group [10]. These studies have found that the overall incidence of solid tumours is rising in the area. This is a trend which is in agreement with a number of other studies from other parts of Europe and elsewhere in the world [7,11-14].

The present study updates the previous analyses from the Northern Region of England and, more specifically examines sex-specific trends in the incidence of individual diagnostic groups. It was hypothesized that diagnostic groups that show only marginal or non-statistically significant results when the sexes are pooled would show more distinct and significant results when examined by sex.

The aim of this study was to determine whether there were sex-specific trends in the incidence of solid tumours in the Northern Region of England. It is well known that cancer patterns are substantially different between children and the group comprising adolescents and young adults. Thus, analyses were stratified by age-group (0 – 14 year olds and 15 – 24 year olds).

## Methods

### Study Subjects

All cases of solid tumours in 0 – 24 year olds diagnosed in the Northern Region of England during the period 1968 – 2005 were obtained from the Northern Region Young Persons' Malignant Disease Registry (NRYPMDR). The NRYPMDR is a specialist cancer registry covering the counties of Northumberland, Tyne and Wear, Durham, Teesside and Cumbria (excluding Barrow-in-Furness). All cases of malignant disease in the region occurring in residents aged under-25 are reported to the registry. The entire region contains about 17% of the under-25 population of England. Tyne and Wear is the 6^th ^largest conurbation in the country and the largest population centre in the study area. The population of the Northern Region is ethnically homogeneous, with fewer than 2% from ethnic minorities [15-17]. Less than 3% born of its population was born outside of the UK, the lowest of any region in England and Scotland, and is similar to Wales [18].

Cases aged 0–14 years have been collected prospectively since 1968. Cases aged 15–24 years have been collected retrospectively for cases diagnosed during 1968–1985 and prospectively since 1985. Cases are identified from multiple sources. Consultants throughout the region notify the registry of any malignancies in children and young adults. Death certificates and hospital admissions are regularly examined. Data are thoroughly cross-checked with regional and national cancer registries at regular intervals. This ensures that information is highly accurate and complete. The same rigorous procedures were applied to the retrospective collection of data on 15–24 year olds for the period 1968–1985. Overall, it has been estimated that ascertainment of cases in 0–24 year olds is greater than 98% [9,19]. The International Classification of Diseases for Oncology (ICDO-2) was used for coding of morphology and primary site of diagnosis [20]. Cases were grouped using a modification of the International Classification of Childhood Cancer (ICCC) [21].

### Statistical Methods

Incidence rates per million population were calculated based on mid-year population estimates for the study region obtained from the Office for National Statistics. Age-standardized rates (ASRs) and 95% confidence intervals (CIs) were calculated based on a standard world population [22]. ASRs (and 95% CIs) were calculated separately for males and females in childhood (aged 0–14) and adolescence/young adulthood (aged 15–24). This was done for the entire study period (1968–2005) and also for three shorter time periods (1968–1980, 1981–1993, 1994–2005) to assess temporal variation. Temporal trends in annual ASRs were analysed using a linear regression model that included year as a covariate. Furthermore, a significance test for a male/female difference in trend was obtained by including covariates year, sex and the interaction term year* sex in a linear regression model. Statistical significance was taken as *P *< 0.05.

## Results

There were a total of 3576 cases of primary solid tumour diagnosed during the period 1968–2005. 1843 were in males, of whom 886 were aged 0–14 and 957 were aged 15–24. 1733 were in females, of whom 791 were aged 0–14 and 942 were aged 15–24. Overall numbers of cases and age-standardized rates (per million population) with 95% confidence intervals (CIs) by diagnosis, age-group and sex, are given for the entire time period (1968–2005) in Table 1. Solid tumours in males aged 0–14 years were dominated by central nervous system (CNS) tumours (43.1%), with soft tissue sarcomas (14.3%) and sympathetic nervous system tumours (11.5%) being the next most predominant groups, whilst for males aged 15–24 germ cell tumours (30.5%), CNS tumours (23.4%) and carcinomas (23.1%) were the largest diagnostic groups. 27.6% of the male carcinomas in the 15–24 year old group were melanomas. For females aged 0–14 years CNS tumours (39.1%), sympathetic nervous system tumours (12.0%) and renal tumours (10.7%) were the three most common types of solid tumour. However, females aged 15–24 years presented a contrasting spectrum to males from this age-group. Carcinomas (50.1%) dominated the diagnoses, followed by CNS tumours (17.5%) and germ cell tumours (13.5%) in these ages. Carcinomas in females aged 15–24 years included large proportions of melanomas (31.4%) and thyroid cancer (12.9%). A large proportion of the carcinomas (18.0%) were also tumours in the genital and other reproductive areas, and 8.9% of carcinomas were breast tumours.

**Table 1:** Numbers of cases and age-standardized rates (per million population) with 95% confidence intervals (CIs) by diagnosis, age-group and sex, 1968–2005

		**Ages 0–14**		**Ages 15–24**	
		**No.**	**Rate (95% CI)**	**No.**	**Rate (95% CI)**
**CNS tumours**	M	382	31.7 (28.5,35.0)	224	27.3 (23.7,30.9)
	F	309	27.6 (24.4,30.7)	165	20.2 (17.1,23.3)
Ependymomas	M	51	4.6 (3.3,5.8)	21	2.6 (1.6,3.9)
	F	21	2.0 (1.2,3.1)	6	0.8 (0.3,1.6)
Astrocytomas	M	137	11.1 (9.2,12.9)	76	9.3 (7.2,11.4)
	F	126	11.2 (9.2,13.1)	54	6.6 (4.8,8.4)
PNET	M	78	6.7 (5.2,8.2)	13	1.6 (0.8,2.7)
	F	45	4.2 (3.0,5.4)	7	0.9 (0.4,1.8)
Other gliomas	M	34	2.9 (1.9,3.8)	36	4.3 (2.9,5.8)
	F	34	3.0 (2.0,4.0)	26	3.2 (2.1,4.7)
**Sympathetic nervous system tumours**	M	102	9.8 (7.9,11.7)	9	1.1 (0.5,2.1)
	F	95	9.7 (7.8,11.7)	9	1.1 (0.5,2.2)
**Retinoblastoma**	M	38	3.9 (2.6,5.1)	0	-
	F	50	5.4 (3.9,6.8)	0	-
**Renal tumours**	M	71	6.8 (5.2,8.4)	8	0.9 (0.4,1.8)
	F	85	8.7 (6.9,10.6)	7	0.8 (0.3,1.7)
**Hepatoblastomas**	M	7	0.7 (0.3,1.5)	0	-
	F	10	1.0 (0.5,1.9)	2	0.2 (0.0,0.8)
**Bone tumours**	M	65	4.7 (3.5,5.8)	114	14.2 (11.6,16.8)
	F	56	4.3 (3.2,5.5)	66	8.3 (6.3,10.3)
Osteosarcoma	M	34	2.4 (1.6,3.3)	60	7.5 (5.6,9.5)
	F	37	2.8 (1.9,3.8)	39	4.9 (3.4,6.5)
Ewing's sarcoma	M	29	2.1 (1.4,3.0)	36	4.5 (3.0,5.9)
	F	16	1.3 (0.7,2.1)	16	2.0 (1.2,3.3)
**Soft tissue sarcomas**	M	127	11.0 (9.1,13.0)	89	10.9 (8.6,13.2)
	F	72	6.2 (4.8,7.7)	94	11.7 (9.3,14.0)
Rhabdomyosarcoma	M	75	6.8 (5.3,8.4)	14	1.8 (1.0,3.0)
	F	39	3.6 (2.5,4.7)	21	2.7 (1.6,4.1)
Other	M	52	4.2 (3.0,5.4)	75	9.1 (7.1,11.2)
	F	33	2.6 (1.7,3.5)	73	9.0 (6.9,11.1)
**Germ cell tumours**	M	55	5.2 (3.8,6.6)	292	34.8 (30.8,38.8)
	F	60	5.5 (4.1,6.9)	127	15.4 (12.7,18.1)
Gonadal	M	32	3.2 (2.1,4.3)	260	31.0 (27.2,34.7)
	F	18	1.4 (0.8,2.2)	86	10.4 (8.2,12.6)
Non-gonadal	M	23	2.0 (1.3,3.0)	32	3.8 (2.5,5.2)
	F	42	4.1 (2.9,5.4)	41	5.0 (3.5,6.6)
**Carcinomas**	M	39	2.9 (2.0,3.9)	221	26.4 (22.9,29.9)
	F	54	4.5 (3.3,5.7)	472	56.5 (51.4,61.6)
Thyroid	M	9	0.7 (0.3,1.3)	23	2.8 (1.7,4.1)
	F	9	0.7 (0.3,1.4)	61	7.4 (5.6,9.3)
Melanoma	M	7	0.5 (0.2,1.1)	61	7.4 (5.5,9.2)
	F	16	1.4 (0.8,2.3)	148	17.9 (15.0,20.7)
Skin	M	4	0.3 (0.1,0.7)	39	4.6 (3.1,6.0)
	F	4	0.3 (0.1,0.7)	43	5.2 (3.6,6.7)
Breast	M	0	-	1	0.1 (0.0,0.7)
	F	0	-	42	4.9 (3.4,6.4)
Genital/other reproductive	M	0	-	0	-
	F	1	0.1 (0.0,0.6)	85	9.9 (7.8,12.0)
Lung and bronchial	M	1	0.1 (0.0,0.4)	7	0.8 (0.3,1.7)
	F	2	0.1 (0.0,0.5)	5	0.6 (0.2,1.4)
Colorectal	M	0	-	19	2.3 (1.4,3.6)
	F	2	0.1 (0.0,0.5)	9	1.1 (0.5,2.0)

**TOTAL**	M	886	76.8 (71.7,81.9)	957	115.7 (108.3,123.0)
	F	791	73.0 (67.8,78.1)	942	114.3 (107.0,121.6)

The results of the analyses of trends are shown in Tables 2 and 3. For 0–14 year olds, there were overall statistically significant increases for both sexes. There was also an increase in incidence for both male and female CNS tumours in 0–14 year olds (males: 1.5% per year; 95% CI: 0.5–2.5 and females: 1.2% per year; 95% CI: 0.2%–2.2%). This was due to increases in the incidence of astrocytoma (males: 3.1% per year; 95% CI: 1.7%–4.6% and females: 4.4% per year; 95% CI: 2.3%–6.6%). There were a number of sex-specific incidence trends for 0–14 year olds. Male-specific increases were observed for rhabdomyosarcoma (average annual increase of 2.4%, 95% CI: 0.2%–4.5%), carcinomas overall (3.2% per year, 95% CI: 0.1%–6.3%) and non-melanotic skin cancer (9.6% per year, 95% CI: 0.0%–19.2%). There were female-specific increases for sympathetic nervous system tumours (2.2% per year; 95% CI: 0.4%–3.9%), gonadal germ cell tumours (8.6% per year; 95% CI: 4.3%–12.9%) and non-gonadal germ cell tumours (5.4% per year; 95% CI: 2.8%–7.9%).

**Table 2:** Numbers of cases aged 0–14 years, age-standardized rates (per million population) and 95% CIs, average annual percentage rate changes and 95% CIs, by diagnosis, sex and time period and test for sex-difference in temporal trend (*P*-value)

			1968–1980		1981–1993		1994–2005	% Ann. Inc	M/F diff
**CNS tumours**	Male	134	28.5 (23.6,33.4)	115	29.9 (24.4,35.4)	133	38.4 (31.8,45.0)	1.5 (0.5,2.5)	0.74
	Female	116	26.2 (21.3,31.0)	91	25.1 (19.9,30.3)	102	32.7 (26.3,39.2)	1.2 (0.2,2.2)	
Ependymomas	Male	18	4.3 (2.5,6.8)	17	4.7 (2.7,7.5)	16	4.9 (2.8,8.0)	0.7 (-1.7,3.2)	0.61
	Female	10	2.4 (1.2,4.5)	6	1.6 (0.6,3.5)	5	1.9 (0.6,4.5)	-0.5 (-4.6,3.6)	
Astrocytomas	Male	38	7.9 (5.4,10.5)	44	11.1 (7.8,14.4)	55	15.5 (11.3,19.6)	3.1 (1.7,4.6)	0.33
	Female	36	8.1 (5.4,10.8)	29	7.9 (5.3,11.4)	61	19.5 (14.5,24.5)	4.4 (2.3,6.6)	
PNET	Male	35	7.5 (4.9,10.0)	16	4.4 (2.5,7.2)	27	8.1 (5.3,11.8)	0.6 (-1.6,2.9)	0.86
	Female	16	3.8 (2.1,6.1)	15	4.3 (2.4,7.1)	14	4.7 (2.5,7.8)	0.9 (-1.7,3.6)	
Other gliomas	Male	13	2.9 (1.5,5.0)	10	2.6 (1.2,4.7)	11	3.1 (1.5,5.6)	0.7 (-2.4,3.9)	0.42
	Female	15	3.4 (1.9,5.6)	9	2.6 (1.2,4.9)	10	2.9 (1.4,5.4)	-1.0 (-3.7,1.7)	
**Sympathetic nervous system tumours**	Male	32	7.4 (4.8,10.0)	43	12.6 (8.9,16.4)	27	9.6 (6.3,14.0)	0.9 (-0.5,2.4)	0.29
	Female	25	6.9 (4.5,10.2)	34	10.3 (6.8,13.8)	36	13.3 (8.9,17.6)	2.2 (0.4,3.9)	
**Retinoblastoma**	Male	16	4.2 (2.4,6.8)	12	3.6 (1.9,6.3)	10	3.6 (1.7,6.6)	-0.3 (-3.6,3.1)	0.42
	Female	26	7.2 (4.7,10.5)	17	5.4 (3.1,8.6)	7	2.7 (1.1,5.6)	-2.1 (-5.0,0.8)	
**Renal tumours**	Male	28	6.8 (4.5,9.9)	18	5.1 (3.0,8.0)	25	9.0 (5.8,13.3)	0.5 (-1.5,2.6)	0.86
	Female	31	8.3 (5.3,11.2)	29	8.8 (5.9,12.7)	25	9.4 (6.1,13.9)	0.3 (-1.5,2.1)	
**Hepatoblastomas**	Male	1	0.3 (0.0,1.5)	1	0.3 (0.0,1.7)	5	1.9 (0.6,4.4)	7.3 (-0.2,14.9)	0.18
	Female	5	1.3 (0.4,3.0)	2	0.6 (0.1,2.3)	3	1.2 (0.2,3.4)	0.5 (-5.5,6.6)	
**Bone tumours**	Male	30	5.3 (3.4,7.2)	17	4.0 (2.3,6.4)	18	4.6 (2.7,7.3)	0.3 (-2.1,2.7)	0.58
	Female	21	4.1 (2.5,6.3)	21	5.1 (3.1,7.8)	14	3.8 (2.1,6.4)	-0.7 (-3.3,1.9)	
Osteosarcoma	Male	18	3.1 (1.9,5.0)	8	1.9 (0.8,3.8)	8	2.0 (0.9,3.9)	-0.9 (-4.3,2.6)	0.80
	Female	16	3.1 (1.8,5.1)	12	2.9 (1.5,5.0)	9	2.5 (1.1,4.7)	-1.5 (-4.2,1.3)	
Ewing's sarcoma	Male	12	2.2 (1.1,3.8)	9	2.0 (0.9,3.9)	8	2.0 (0.9,4.0)	0.3 (-2.4,3.0)	0.94
	Female	4	0.8 (0.2,2.2)	9	2.2 (1.0,4.2)	3	0.8 (0.2,2.4)	0.1 (-4.9,5.1)	
**Soft tissue sarcomas**	Male	45	9.6 (6.7,12.4)	46	12.5 (8.9,16.2)	36	11.2 (7.5,14.9)	1.3 (-0.2,2.8)	0.70
	Female	28	6.0 (4.0,8.7)	22	5.8 (3.6,8.8)	22	7.0 (4.4,10.7)	0.8 (-1.2,2.9)	
Rhabdomyosarcoma	Male	24	5.4 (3.4,8.1)	26	7.2 (4.7,10.6)	25	8.3 (5.3,12.3)	2.4 (0.2,4.5)	0.09
	Female	19	4.3 (2.5,6.7)	10	2.7 (1.3,5.1)	10	3.7 (1.7,6.7)	-0.4 (-2.7,1.9)	
Other	Male	21	4.2 (2.6,6.4)	20	5.3 (3.2,8.2)	11	2.9 (1.4,5.2)	-0.4 (-2.9,2.1)	0.19
	Female	9	1.7 (0.8,3.3)	12	3.1 (1.6,5.4)	12	3.4 (1.7,5.9)	2.5 (-1.1,6.0)	
**Germ cell tumours**	Male	17	4.4 (2.5,7.0)	18	5.1 (3.0,8.1)	20	6.5 (4.0,10.2)	2.1 (-0.7,4.8)	0.02
	Female	8	1.8 (0.7,3.5)	20	5.7 (3.5,8.9)	32	10.6 (6.9,14.4)	6.2 (4.1,8.3)	
Gonadal	Male	13	3.4 (1.8,5.8)	13	3.9 (2.1,6.6)	6	2.1 (0.8,4.6)	-1.1 (-4.9,2.7)	0.001
	Female	2	0.4 (0.0,1.3)	2	0.5 (0.1,1.8)	14	4.0 (2.1,6.7)	8.6 (4.3,12.9)	
Non-gonadal	Male	4	1.0 (0.3,2.5)	5	1.2 (0.4,2.9)	14	4.4 (2.4,7.5)	7.2 (2.6,11.8)	0.50
	Female	6	1.4 (0.5,3.1)	18	5.2 (3.1,8.3)	18	6.6 (3.9,10.5)	5.4 (2.8,7.9)	
**Carcinomas**	Male	11	2.0 (1.0,3.7)	13	3.0 (1.6,5.1)	15	4.2 (2.3,6.9)	3.2 (0.1,6.3)	0.31
	Female	17	3.8 (2.2,6.1)	20	5.0 (3.0,7.7)	17	5.0 (2.9,8.0)	1.1 (-1.5,3.7)	
Thyroid	Male	2	0.4 (0.0,1.3)	2	0.5 (0.1,1.7)	5	1.3 (0.4,3.0)	5.9 (-0.4,12.2)	0.07
	Female	4	0.9 (0.2,2.4)	3	0.7 (0.1,2.0)	2	0.6 (0.1,2.0)	-1.8 (-7.1,3.4)	
Melanoma	Male	2	0.3 (0.0,1.2)	3	0.7 (0.1,2.0)	2	0.6 (0.1,2.3)	4.3 (-2.8,11.4)	0.81
	Female	3	0.7 (0.1,2.2)	8	2.1 (0.9,4.1)	5	1.6 (0.5,3.7)	3.1 (-2.7,9.0)	
Skin	Male	0	-	2	0.4 (0.1,1.5)	2	0.5 (0.1,1.9)	9.6 (0.0,19.2)	0.46
	Female	0	-	2	0.5 (0.1,1.6)	2	0.5 (0.1,1.8)	4.5 (-4.8,13.8)	
Breast	Male	0	-	0	-	0	-	-	-
	Female	0	-	0	-	0	-	-	
Genital/other reproductive	Male	0	-	0	-	0	-	-	-
	Female	0	-	1	0.3 (0.0,1.8)	0	-	2.9 (-15.1,20.9)	
Lung and bronchial	Male	1	0.2 (0.0,1.1)	0	-	0	-	-12.1 (-25.9,1.6)	0.12
	Female	1	0.2 (0.0,1.0)	0	-	1	0.3 (0.0,1.4)	2.8 (-9.6,15.2)	
Colorectal	Male	0	-	0	-	0	-	-	-
	Female	1	0.2 (0.0,1.0)	0	-	1	0.3 (0.0,1.4)	2.5 (-9.9,14.8)	

**TOTAL**	Male	314	68.6 (60.8,76.3)	283	76.1 (67.1,85.0)	289	89.0 (78.6,99.4)	1.3 (0.7,1.9)	0.84
	Female	277	65.4 (57.6,73.2)	256	71.8 (62.9,80.7)	258	85.7 (75.0,96.3)	1.2 (0.5,2.0)	

Figures are counts, ASRs with 95% CIs, Annual Percentage Change in ASR with 95% CIs (estimated from linear regression model) and p value for testing M/F difference in trend (obtained from year *sex interaction term in linear regression model)

**Table 3:** Numbers of cases aged 15–24 years, age-standardized rates (per million population) and 95% CIs, average annual percentage rate changes and 95% CIs, by diagnosis, sex and time period and test for sex-difference in temporal trend (*P*-value)

			1968–1980		1981–1993		1994–2005	% Ann Inc	M/F diff
**CNS tumours**	Male	87	29.0 (22.9,35.1)	84	28.4 (22.3,34.5)	53	23.6 (17.3,30.0)	-0.5 (-1.9,0.8)	0.60
	Female	59	19.8 (14.7,24.8)	59	20.2 (15.1,25.4)	47	20.8 (14.9,26.8)	0.0 (-1.5,1.5)	
Ependymomas	Male	8	2.7 (1.2,5.3)	10	3.4 (1.6,6.3)	3	1.3 (0.3,3.9)	-2.1 (-5.5,1.4)	0.19
	Female	2	0.7 (0.1,2.5)	1	0.4 (0.0,2.1)	3	1.3 (0.3,3.9)	3.2 (-3.9,10.3)	
Astrocytomas	Male	28	9.4 (6.2,13.6)	25	8.6 (5.6,12.7)	23	10.2 (6.5,15.3)	0.4 (-1.9,2.7)	0.71
	Female	21	7.0 (4.4,10.8)	17	5.8 (3.4,9.3)	16	7.1 (4.0,11.5)	-0.2 (-2.4,2.0)	
PNET	Male	8	2.6 (1.1,5.2)	3	1.0 (0.2,2.9)	2	0.9 (0.1,3.2)	-2.8 (-7.9,2.2)	0.99
	Female	4	1.4 (0.4,3.5)	2	0.7 (0.1,2.5)	1	0.5 (0.0,2.6)	-2.9 (-8.8,3.1)	
Other gliomas	Male	14	4.6 (2.5,7.8)	17	5.6 (3.3,9.0)	5	2.2 (0.7,5.2)	-1.4 (-4.4,1.5)	0.99
	Female	11	3.6 (1.8,6.5)	10	3.6 (1.7,6.7)	5	2.2 (0.7,5.1)	-1.4 (-5.3,2.4)	
**Sympathetic nervous system tumours**	Male	1	0.3 (0.0,1.8)	6	2.1 (0.8,4.6)	2	0.9 (0.1,3.2)	1.5 (-4.1,7.0)	0.71
	Female	2	0.7 (0.1,2.4)	3	1.1 (0.2,3.1)	4	1.9 (0.5,4.8)	3.1 (-3.4,9.7)	
**Retinoblastoma**	Male	0	-	0	-	0	-	-	-
	Female	0	-	0	-	0	-	-	
**Renal tumours**	Male	1	0.3 (0.0,1.8)	3	1.0 (0.2,2.9)	4	1.7 (0.5,4.3)	2.8 (-4.0,9.6)	0.32
	Female	3	1.0 (0.2,3.0)	3	0.9 (0.2,2.7)	1	0.4 (0.0,2.4)	-1.8 (-7.9,4.3)	
**Hepatoblastomas**	Male	0	-	0	-	0	-	-	-
	Female	1	0.3 (0.0,1.8)	1	0.3 (0.0,1.7)	0	-	-4.4 (-16.0,7.3)	
**Bone tumours**	Male	36	12.1 (8.2,16.1)	50	17.7 (12.8,22.6)	28	12.6 (8.4,18.3)	0.2 (-1.7,2.0)	0.15
	Female	19	6.4 (3.9,10.0)	22	7.9 (4.9,11.9)	25	11.6 (7.5,17.1)	2.2 (0.2,4.2)	
Osteosarcoma	Male	20	6.8 (4.2,10.6)	23	8.2 (5.2,12.3)	17	7.7 (4.5,12.3)	0.1 (-2.6,2.8)	0.10
	Female	10	3.4 (1.6,6.2)	12	4.3 (2.2,7.5)	17	7.9 (4.6,12.7)	3.5 (0.5,6.5)	
Ewing's sarcoma	Male	9	3.0 (1.4,5.7)	20	7.0 (4.3,10.8)	7	3.2 (1.3,6.6)	1.0 (-1.9,4.0)	0.50
	Female	6	2.1 (0.8,4.5)	5	1.7 (0.6,4.1)	5	2.3 (0.8,5.4)	-0.7 (-4.8,3.4)	
**Soft tissue sarcomas**	Male	27	9.1 (6.0,13.3)	29	9.9 (6.6,14.2)	33	14.6 (9.6,19.6)	1.9 (-0.4,4.2)	0.55
	Female	27	9.2 (6.1,13.4)	39	13.3 (9.1,17.5)	28	12.7 (8.4,18.4)	1.0 (-0.8,2.9)	
Rhabdomyosarcoma	Male	5	1.7 (0.6,4.0)	2	0.7 (0.1,2.6)	7	3.2 (1.3,6.6)	3.0 (-1.5,7.5)	0.74
	Female	4	1.4 (0.4,3.6)	13	4.6 (2.5,7.9)	4	1.8 (0.5,4.5)	2.0 (-2.2,6.1)	
Other	Male	22	7.4 (4.6,11.2)	27	9.1 (6.0,13.3)	26	11.4 (7.5,16.7)	1.7 (-1.0,4.4)	0.60
	Female	23	7.8 (4.9,11.7)	26	8.7 (5.7,12.7)	24	10.9 (7.0,16.3)	0.7 (-1.7,3.2)	
**Germ cell tumours**	Male	70	23.1 (17.7,28.6)	115	37.5 (30.6,44.4)	107	46.5 (37.7,55.3)	2.1 (0.6,3.7)	0.74
	Female	44	14.6 (10.3,18.9)	35	11.6 (7.8,15.5)	48	21.6 (15.5,27.7)	1.7 (-0.2,3.6)	
Gonadal	Male	65	21.5 (16.2,26.7)	101	32.8 (26.4,39.3)	94	40.9 (32.6,49.1)	1.9 (0.3,3.4)	0.51
	Female	29	9.6 (6.4,13.8)	26	8.6 (5.6,12.7)	31	13.8 (9.0,18.7)	1.0 (-1.0,3.0)	
Non-gonadal	Male	5	1.7 (0.5,3.9)	14	4.6 (2.5,7.8)	13	5.6 (3.0,9.6)	4.4 (1.1,7.7)	0.65
	Female	15	5.0 (2.8,8.2)	9	3.0 (1.4,5.7)	17	7.8 (4.5,12.4)	3.2 (-0.7,7.1)	
**Carcinomas**	Male	78	25.7 (20.0,31.4)	75	24.6 (19.0,30.2)	68	29.7 (22.6,36.8)	0.2 (-1.4,1.8)	0.07
	Female	131	43.1 (35.7,50.5)	164	53.9 (45.6,62.1)	177	78.2 (66.7,89.7)	2.2 (0.7,3.7)	
Thyroid	Male	6	2.0 (0.7,4.3)	8	2.8 (1.2,5.5)	9	3.9 (1.8,7.4)	2.5 (-2.2,7.2)	0.90
	Female	13	4.4 (2.3,7.5)	22	7.4 (4.6,11.3)	26	11.5 (7.5,16.9)	2.8 (0.1,5.6)	
Melanoma	Male	20	6.7 (4.1,10.4)	22	7.2 (4.5,10.9)	19	8.3 (5.0,13.0)	0.6 (-2.1,3.3)	0.03
	Female	25	8.3 (5.3,12.2)	54	17.8 (13.0,22.5)	69	30.8 (23.5,38.1)	4.6 (2.2,7.1)	
Skin	Male	9	2.9 (1.3,5.5)	9	2.9 (1.3,5.5)	21	9.1 (5.6,13.8)	4.7 (0.5,8.9)	0.61
	Female	11	3.6 (1.8,6.5)	12	4.1 (2.1,7.1)	20	8.8 (5.4,13.6)	3.2 (-0.4,6.9)	
Breast	Male	0	-	0	-	1	0.5 (0.0,2.6)	11.8 (-8.4,32.0)	0.25
	Female	14	4.5 (2.5,7.6)	18	5.7 (3.4,9.0)	10	4.3 (2.1,8.0)	-0.3 (-2.9,2.3)	
Genital/other reproductive	Male	0	-	0	-	0	-	-	-
	Female	31	10.0 (6.5,13.5)	28	8.8 (5.9,12.8)	26	11.3 (7.4,16.6)	0.5 (-1.7,2.8)	
Lung and bronchial	Male	4	1.3 (0.4,3.4)	3	1.0 (0.2,2.9)	0	-	-6.2 (-12.5,0.2)	0.23
	Female	2	0.7 (0.1,2.4)	1	0.3 (0.0,1.7)	2	0.9 (0.1,3.3)	0.0 (-7.7,7.8)	
Colorectal	Male	10	3.3 (1.6,6.1)	5	1.7 (0.6,4.0)	4	1.7 (0.5,4.4)	-3.5 (-7.4,0.4)	0.01
	Female	1	0.3 (0.0,1.8)	3	1.0 (0.2,2.9)	5	2.2 (0.7,5.0)	8.2 (0.1,16.3)	

**TOTAL**	Male	300	99.8 (88.5,111.1)	362	121.1 (108.6,133.6)	295	129.7 (114.9,144.5)	0.8 (-0.2,1.8)	0.30
	Female	286	95.2 (84.1,106.2)	326	109.2 (97.3,121.1)	330	147.2 (131.3,163.1)	1.6 (0.5,2.7)	

Figures are counts, ASRs with 95% CIs, Annual Percentage Change in ASR with 95% CIs (estimated from linear regression model) and p value for testing M/F difference in trend (obtained from year *sex interaction term in linear regression model)

For 15–24 year olds, there was an overall statistically significant increase for females, but not males. There were male-specific increases in gonadal germ cell tumours (1.9% per year, 95% CI: 0.3%–3.4%), non-gonadal germ cell tumours (4.4% per year, 95% CI: 1.1%–7.7%) and non-melanotic skin cancer (4.7% per year, 95% CI: 0.5%–8.9%). There were female-specific increases for osteosarcoma (3.5% per year; 95% CI: 0.5%–6.5%), carcinomas overall (2.2% per year; 95% CI: 0.7%–3.7%), thyroid cancer (2.8% per year; 95% CI: 0.1%–5.6%) and melanoma (4.6% per year; 95% CI: 2.2%–7.1%).

There were statistically significant differences in temporal trends between the sexes for gonadal germ cell tumours in 0–14 year olds (*P *= 0.001) and melanoma in 15–24 year olds (*P *= 0.03). There was also a statistically significant difference for colorectal tumours in 15–24 year olds (*P *= 0.01).

## Discussion

There was an overall significant increase in the incidence of childhood solid tumours within this region. This finding was consistent with similar data from other countries [12]. Long-term temporal trends, reported in studies from North West England (an area geographical adjacent to the Northern Region), have also found similar results in the under-15 population [13,14]. However many similar papers have reported incidence patterns and temporal trends but have not reported results stratified by sex. Our analyses showed sex-specific differences both in the incidence and trends of certain diagnostic types. Most notably in the 15–24 year olds there were major difference in the distribution of diagnoses between males and females. Carcinomas exhibited a marked female excess in this age-group, which was due to the female preponderance of melanoma, thyroid and breast carcinoma. Furthermore, the overall rise in incidence in the adolescent and young adult group was confined to females.

There were male-specific increases in the incidence of rhabdomyosarcoma (childhood cases), gonadal and non-gonadal germ cell tumours (adolescent and young adult cases) and non-melanotic skin cancer (all ages). There were female-specific increases in the incidence of sympathetic nervous system tumours, gonadal and non-gonadal germ cell tumours (childhood cases), osteosarcoma, thyroid cancer and melanoma (adolescent and young adult cases). Sex-related differences in exposure or response to an aetiological agent that is increasing in prevalence may be predicted to lead to sex-specific differentials in secular trends.

Whilst the increase in the incidence of rhabdomyosarcoma was based on small numbers of cases, an increase in the incidence of rhabdomyosarcoma has also been reported by other studies [7,23]. The reason for the increase is not clear. The aetiology of rhabdomyosarcoma is likely to be different from the other types of soft tissue sarcoma (which generally present at older ages). A number of environmental exposures, including pesticides, dioxin, chlorophenol and benzene, have been linked particularly with increased risk of other types of soft tissue sarcoma [24-28]. The present finding, although based on small numbers, indicates the possibility of sex-linked differences in aetiology for rhabdomyosarcoma.

Rises in the incidence of sympathetic nervous system tumours (mainly neuroblastoma) have been found in both males and females in other studies [7]. However, the female-specific increase is consistent with a similar finding that was reported from the Manchester Children's Tumour Registry [14]. Increased risk of neuroblastoma has been associated with prenatal and perinatal risk factors. These have included gestational diabetes and maternal use of medication and hair dyes during pregnancy [29-31]. The finding from the present study indicates that there may be differential susceptibility to early exposures between males and females.

Thyroid cancer exhibits a marked female excess and this is true in cases among young people [32]. In England and Wales the ratio of female to male cases is 2.5:1 for those aged 0–14 [12]. The reasons for this excess are not clear, but may be due to greater female susceptibility to a triggering environmental exposure. Our results show a sustained excess and increase in incidence for females, which merits closer examination in the future in order to better understand aetiology.

For malignant melanoma the earlier reported temporal increase [10] has been shown here to be sustained and concentrated within females aged over 14 years. Melanoma has been associated with exposure to UV radiation [33]. Our results show a far higher incidence of melanomas in young adult females than similar males and a strikingly high annual rate of increase in incidence of around 5% in females aged 15–24 years. Considering number of nevi as the best predictor of melanoma occurrence [34], previous studies of European schoolchildren have shown males to have a higher count of small nevi than females [35]. However, our results show a female excess in melanoma. This highlights a sex-specific phenomenon, and suggests females in the region may be exposed to increased UV radiation, perhaps through tanning beds, sunbathing, foreign holidays and mode of clothing.

The findings for gonadal germ cell tumours in males, dominating testicular cancer, are consistent with other studies showing that the incidence has risen dramatically elsewhere in the developed world [36,37]. A previous report from this region highlighted this increase and noted that 80% of the tumours are non-seminomas, which tend to peak at earlier ages [38]. Whilst some increase in incidence may be explained by improvement in diagnosis and registration [39], the magnitude and consistency of the increase seen in many industrialized countries suggests other factors, such as socioeconomic status [40], maternal chemical exposures [41], or pre- and postnatal exposure to endogenous oestrogens [42] could play a role.

The marked increases found for female non-gonadal germ cell tumours in 0–14 year olds and for male non-gonadal germ cell tumours in 15–24 year olds were based on small numbers. However, an overall increase (for males and females combined) in 0–14 year olds has been reported previously from the Manchester Children's Tumour Registry [14].

The incidence of CNS tumours, especially astrocytomas, increased amongst both girls and boys aged 0–14 years. Similar increases in the incidence of childhood CNS tumours, especially astrocytomas, have been reported from other studies [14,43,44]. The possible role of artefact should be acknowledged. The upward trend may be due to improvements in registration as well as increases in unknown environmental risk factors [45,46].

The findings of increases for osteosarcoma (females), gonadal germ cell tumours (females) and non-melanotic skin cancer (males) were all based on small numbers and should not be over-interpreted. Indeed, it must be stressed that there are certain inherent limitations to the present study. In particular, it should be acknowledged that the analyses are based on limited numbers of cases from one geographical area. Differences between male and female trends were only formally statistically significant for gonadal tumours in children and melanoma in adolescents/young adults. The interaction for colorectal carcinoma was based on small numbers of cases. It will be of great interest to see if similar findings will be reported from analyses that are conducted in other parts of the UK and in other countries.

## Conclusion

Over the 1968 to 2005 time period there have been significant temporal changes in the incidence of certain solid tumours in under-25s in the Northern Region of England. In some cases marked increases have been found that merit further attention to identify possible causes and suggest preventative approaches. We have shown there can be sex-specific changes that may have been obscured or unidentifiable if the sexes were not analysed separately. The differences shown in melanoma, for instance, point towards a possible aetiological link with a sex-related behaviour or exposure.

The causes of different rates may be because of different exposures, as postulated with melanomas, or due to different susceptibilities, as is possibly the case with thyroid cancer. Sex-related differences in genetics, exposures, behaviours and treatment exist even in preadolescent populations and it therefore is necessary to always report sex-specific data in both childhood and adolescent/young adult cancers. This will improve our understanding of patterns, trends and aetiology.

## Competing interests

The author(s) declare that they have no competing interests.

## Authors' contributions

BLM, PWJ and RJQM contributed to the design of the study, the writing of the manuscript and the analysis and interpretation of data. MTD, AWC and LP contributed to the writing of the manuscript and the interpretation of data.

## Pre-publication history

The pre-publication history for this paper can be accessed here:


